# An Abdominoplasty Modification for Postpregnancy Abdomen with Rectus Diastasis and Midline Hernia: The Technique and Results

**DOI:** 10.1097/PRS.0000000000010637

**Published:** 2023-05-09

**Authors:** Reetta Tuominen, Hilkka Peltoniemi, Tiina Jahkola, Jaana Vironen

**Affiliations:** Helsinki, Finland; From the 1 Department of Plastic Surgery; 3 Abdominal Center, Helsinki University Hospital and University of Helsinki; 2 Eira Hospital.

## Abstract

**Summary:**

After pregnancy, some women experience severe rectus diastasis (RD), with body control dysfunction, midline hernia, or other quality-of-life impairment. The purpose of this study was to describe the authors’ experience using hydrodissection and epidural anesthesia for lateral plication modification of abdominoplasty to restore abdominal wall firmness. A total of 46 consecutive patients with RD after pregnancy were enrolled. The mean intraoperative inter-rectus distance was 4.6 cm. RD is not always the only structure that has been elongated. Firmness of the abdominal wall also depends on lateral fascia structures. This study reports the total plicated distance addressing the lateral laxity in the abdominal wall. In this series, total plication was 7.8 cm, and 16 patients had a midline hernia. No hernia recurrences occurred, and the rectus bellies were less than 5 mm apart from each other in all participants, verified with ultrasound after 2 years of follow-up. Patient perspective of care and surgical outcome were recorded. Health-related quality-of-life domains were significantly improved postoperatively. Lumbar back pain visual analogue scale score was 4.5 ± 2.3 preoperatively and 0.5 ± 0.9 postoperatively. The ability to perform sit-ups increased from zero to 11, suggesting better motor control. The total complication rate was 10.9%. Hydrodissection and epidural anesthesia for lateral plication modification offers a reliable and effective treatment method for RD repair with and without a small midline hernia with a low complication rate.

**CLINICAL QUESTION/LEVEL OF EVIDENCE:**

Therapeutic, IV.

Hydrodissection and epidural anesthesia for lateral plication (HELP) surgery is performed using epidural anesthesia. The catheter is placed at the T8 through T10 level. First, saline with adrenaline (1 mg/1000 mL) is infiltrated above the fascia layer with Body-jet or with a simple 50-mL syringe and a blunt, long cannula. Hydrodissection reduces bleeding and facilitates dissection. No fat is left on top of the fascia that is to be plicated. Oval, 1 × 1.5-cm umbilicus is dissected circumferentially from the flap, leaving it attached to the abdomen with a short umbilical stalk. In case of a hernia, the hernia sac or protruding preperitoneal fat is dissected from the fascia opening, and the hernia is either returned to the preperitoneal/intraperitoneal space or ligated. The fascia opening is closed temporarily with absorbable sutures. Plication of linea alba is performed in three layers: nonabsorbable multifilament (Ethibond 2; Ethicon) coronal plane figure-of-eight sutures are placed first (Fig. 1), followed by two layers of barbed, thick, slowly absorbable running sutures (Quill 2; Surgical Specialties). The vertical excess is controlled with figure-of-eight sutures; if there is more vertical excess, the figure-of-eight sutures are longer. The knots of nonabsorbable Ethibond interrupted sutures are buried with barbed Quill 2, which is important, especially with very thin patients, who might sensate all irregularities in the fascia layer. The Quill 2 suture has two needles, and the suturing is started at the umbilicus. The other arm of the suture heads into the cranial direction. At the xiphoideum, care must be taken to ensure that the entire divarication and looseness is addressed. After reaching the xiphoid process, the plication is shifted into caudal direction, and a third layer of sutures is performed. Knots are not needed. The other arm of Quill 2 is reached from the umbilicus to symphysis and back again to cranial direction. Plication is performed 0.5 to 1 cm beyond medial borders on rectus muscles, and more if lateral laxity is present. The correct tension of the fascia layer can be tested when the patient lifts the head. [See Video (online), which demonstrates a perioperative summary of a patient with rectus diastasis and lateral laxity before plication, during plication, and after three layers of plication.] Eight to 12 tension sutures are placed from subcutis to fascia to narrow the dead space and to reduce seroma formation. Skin opening for the umbilicus is I-shaped with 2-mm oblique extensions into four directions (1, 5, 7, and 11 o’clock). The umbilicus is repositioned with four absorbable three-point sutures through plicated fascia layer, umbilical stalk, and dermis of the new skin opening that is prepared with removal of the fat. Later, the skin edges of the umbilical stalk and the new opening are adjusted with simple absorbable, monofilament 6-0 sutures. No suction drains are routinely left. The bikini line is sutured in layers as usual. Operative time is between 120 and 180 minutes depending on the patient’s size and the presence of hernia. Bleeding during the operation is minimal at approximately 20 mL. After surgery, an abdominal elastic band is placed and worn 24 hours a day for 2 to 4 weeks, and for 2 to 4 weeks after that time during the day. Discharge is on the first postoperative day. No anticoagulation is used unless the patient has predisposing factors for deep venous thrombosis. Patients wear antiembolic stockings until they are mobilizing properly.

**Video.** This video demonstrates a perioperative summary of a patient with rectus diastasis and lateral laxity before plication, during plication, and after three layers of plication.

**Fig. 1.**
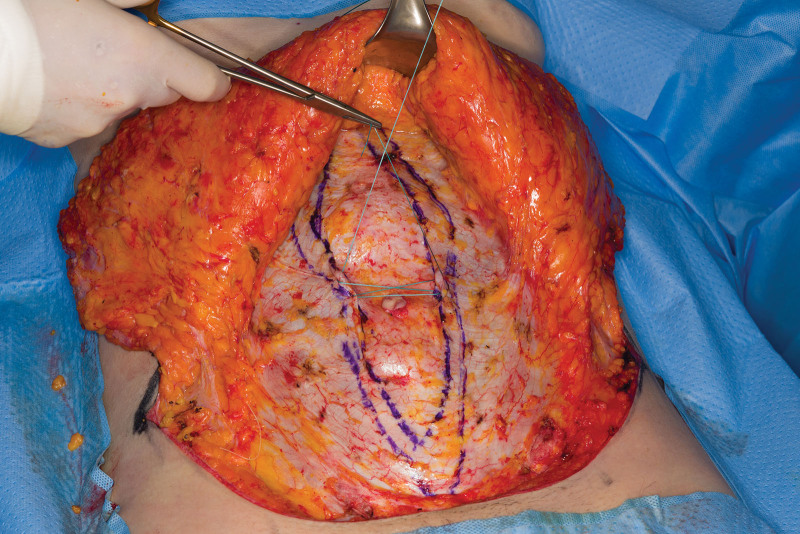
First figure-of-eight suture with Ethibond 0. The more lateral the excess, the more lateral the plication is extended on top of the rectus sheath. Medial borders of the rectus muscles and the plication estimation are marked.

A total of 46 consecutive women with symptomatic rectus diastasis (RD) were enrolled from February 5, 2018, through August 3, 2021. Patient demographic characteristics are presented in Table 1. A patient example is presented in Figure 2. Each participant received study information and completed a written consent form. This study was conducted in accordance with the Declaration of Helsinki and Good Clinical Practice guidelines, and was approved by the regional ethics review board at Helsinki University Hospital (1815/2021). Smoking and excessive visceral obesity were contraindications for surgery.

**Table 1.** Demographic Data

Characteristics	Mean	Minimum	Maximum	No. (Total *n* = 46)
Age, yrs	40.5	32.1	54.9	
Parity	2.0	1	4	
Gemini pregnancies				12
Cesarean deliveries				20
BMI, kg/m^2^	24.0	18.8	33.2	
IRD, cm	4.6	2.3	9.0	
Mild (>2 to ≤3)				2
Moderate (>3 to ≤5)				30
Severe (>5)				14
Total plicated fascia, cm	7.8	6.0	13.5	
Hernia, mm				
Umbilical fascia defect	11.3	5.0	20.0	11^a^
Epigastric fascia defect	12.5	5.0	20.0	6^a^

BMI, body mass index; IRD, inter-rectus diameter.

a One patient had both an epigastric and an umbilical hernia. Thus, the total number of patients with midline hernias was 16 of 46.

**Fig. 2.**
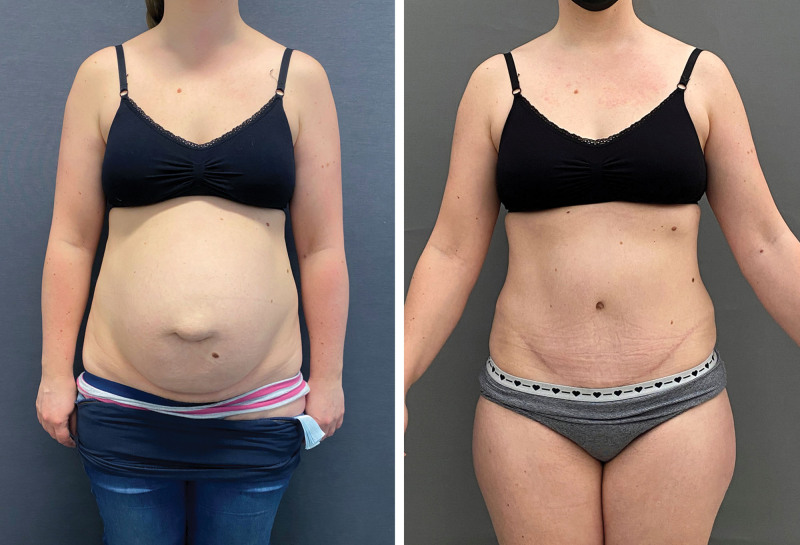
(*Left*) Preoperative photograph of a patient with 7 cm rectus diastasis, 13 cm total laxity, and an umbilical hernia. (*Right*) Postoperative photograph.

As a primary outcome, no hernia recurrences were seen in this series. The rectus bellies were from 0 to 5 mm apart from each other (mean, 1.6 mm). A total of 36 patients (78%) were available for ultrasound measurement, which was performed at a median period of 26.6 months (range, 8 to 46 months) postoperatively.

Health-related quality of life and other measures were assessed as secondary outcomes. There was a significant increase in health-related quality-of-life scores between data points in physical functioning, bodily pain, general health, physical role functioning, social functioning, vitality, and mental health. Preoperative and postoperative RAND-36 scores (mean ± SD) were as follows: physical functioning, 73.8 ± 16.2 and 96.6 ± 5.5 (*P =* 0.00000); bodily pain, 53.3 ± 21.6 and 88.0 ± 14.0 (*P =* 0.00000); general health, 67.8 ± 21.6 and 81.3 ± 17.3 (*P =* 0.0002); role functioning, 68.5 ± 36.7 and 96.2 ± 15.8 (*P =* 0.00005); social functioning, 71.7 ± 24.6 and 93.2 ± 13.6 (*P =* 0.00000); vitality, 42.8 ± 18.0 and 56.5 ± 13.9 (*P =* 0.00001); and mental health, 69.7 ± 17.6 and 78.7 ± 13.9 (*P =* 0.00499), respectively. Before surgery, 56.5% of the participants were unable to perform sit-ups, and the median sit-up score was 0, even though the study group was in general athletic, exercising two to six times a week. Postoperatively, with the same activity level, all but one patient whose back had been operated on previously were able to perform sit-ups, and the median score was 11.5. Postoperative data on sit-ups were missing for six participants.

The total complication rate was 10.8%. Five Clavien-Dindo I complications occurred: two cases of umbilical cellulitis, one small 20-mL seroma without intervention, one bedside-opened local wound infection, and one case of local pain lasting 4 weeks. The pain likely was caused by a tension suture, and it ceased with blunt needle manipulation under local anesthesia to cut the suture.

## DISCUSSION

Fewer than 2% of women develop a wide diastasis after pregnancy.^1^ Patients with RD experience an inability to perform the same activities than before the pregnancy, do sit-ups, lift a child in a rotational torque, or get up from bed sideways, and need to “suck the stomach in” to support the back, leading to a reduction in quality of life. Extensive rehabilitation and postpartum physiotherapy are the primary interventions, but they may not be effective. There are data showing that operative treatment of RD improves functioning and quality of life.^2–5^ In the current study, there was a substantial increase in health-related quality-of-life domains of pain and physical functioning, as well as other domains, after surgery. Patients’ scores were below the age-matched values before HELP abdominoplasty, but above them afterward. Visual analog scale scores measuring back pain declined significantly, from more than 4 to almost 0. In this study, the inability to perform sit-ups preoperatively, even though the participants were exercising two to six times a week, was consistent.

**Fig. 3.**
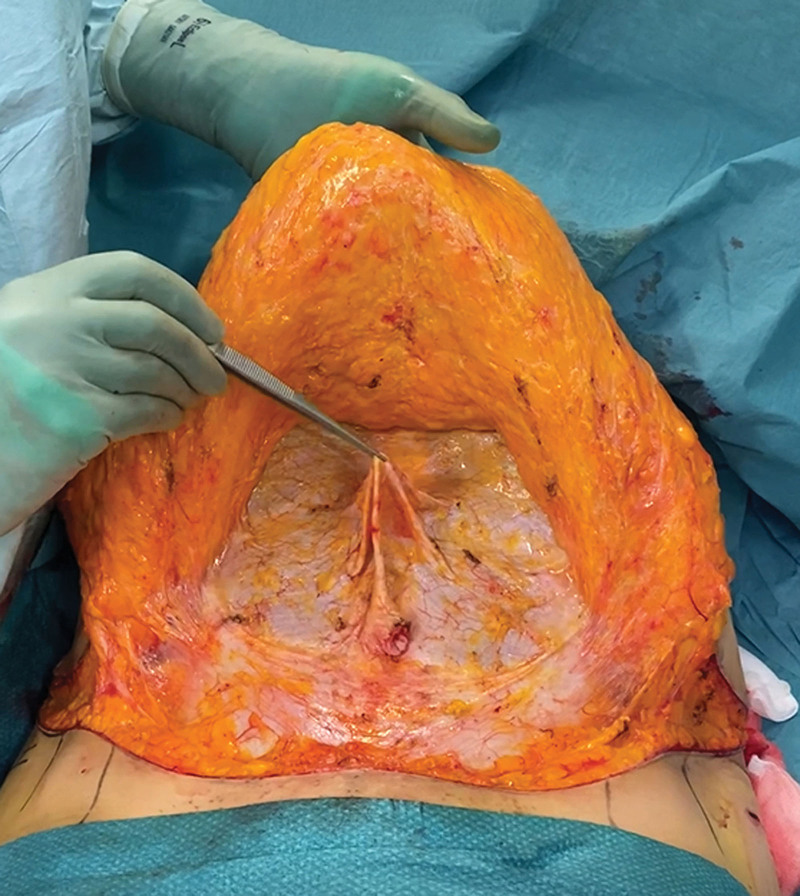
Demonstration of total laxity of the fascia. Hydrodissection creates a clear operating field without bleeding or overlaying fat on top of the fascia layer.

**Fig. 4.**
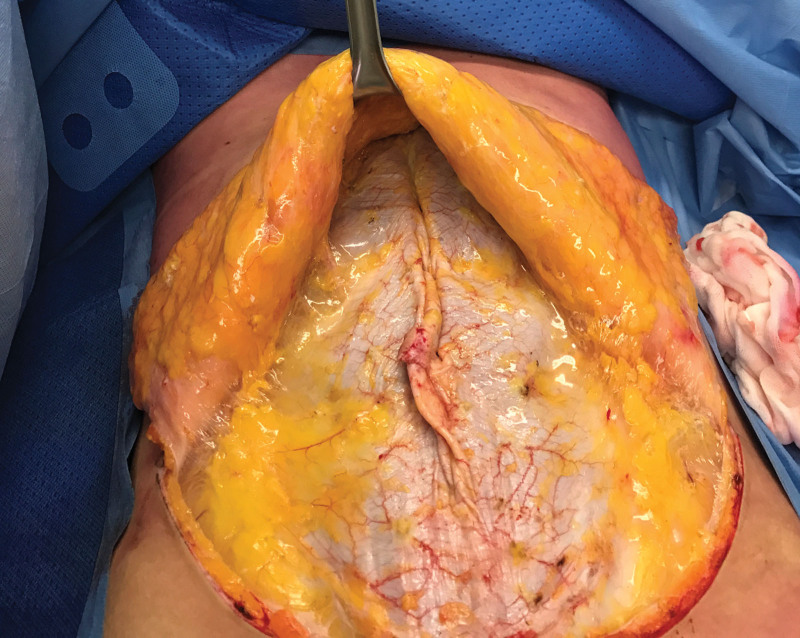
The patient, under epidural anesthesia on the operating table, is asked to lift her head upward. The space between rectus bellies narrows but the loose fascia does not support the musculature.

As the abdominal wall expands during pregnancy, the entire fascia layer stretches, not only the linea alba. After pregnancy, protrusion of the abdominal wall is usually a sign of RD, but also can be caused by lateral laxity, and the actual RD can be mild.^6^ It has been proposed that a loose anterior abdominal wall, and not the RD alone, is a risk factor for core instability and back pain.^7,8^ Patients with RD often have substantial laxity in the fascia layer, as demonstrated in Figures 3 and 4. With the HELP technique, lateral laxity and abdominal bulging are addressed by extending plication as far as necessary beyond the medial borders of the rectus sheaths. This approach theoretically protects the lateral nerves from being trapped into sutures better than the Nahas B-type L-shape fascia plication procedure.^6^ Brauman^7^ controls the vertical excess with sleeve plication at the level of the umbilicus. Both the horizontal and vertical laxity can be addressed by multiple interrupted figure-of-eight sutures.

RD predisposes to midline hernia and is a significant risk factor for hernia recurrence.^9^ Thus, patients with RD are treated by both general and plastic and reconstructive surgeons. Treatment algorithms in these specialties are not the same. In the hernia literature, mesh is suggested if the fascia defect is greater than 1 cm.^10^ The guideline of midline hernia management does not distinguish different subgroups of patients with hernia, so those with diastasis are not addressed separately.^11^ In connection with RD, the hernia is merely a consequence, and this subgroup may benefit from a different approach than a hernia repair. In this homogenous series, no hernia recurrences were seen during the follow-up of 26 months.

The complication rate of abdominoplasty as a sole surgical procedure is approximately 4% to 25%.^12,13^ In the current study with the HELP procedure, the complication rate was 10.8%, with only Clavien-Dindo class I complications observed. In our experience, the infiltration of adrenaline solution to the fascia layer blocks bleeding of minor vessels, minimizes blood loss, and facilitates dissection even in scarred tissues. Epidural anesthesia might reduce the risk of thromboembolism.^14^ The catheter is left in place for the first night to facilitate efficient pain management, low opioid consumption, and immediate mobilization. In some studies, seroma formation is the most common complication.^12^ There is some evidence that the risk of seroma formation may be reduced by using tension sutures.^15^ This has been our experience as well. In this series, there was only one small seroma that resolved without intervention.

RD repair may be considered not only aesthetic but also reconstructive surgery in some cases. Lateral laxity is important to address during surgery. HELP abdominoplasty offers a reliable method with good results and low complication rates for operative treatment of RD with or without a small midline hernia.

## DISCLOSURE

The authors declare that they have no conflicts of interest to disclose.

## PATIENT CONSENT

Patients provided written informed consent for the use of their images.

## ACKNOWLEDGMENTS

Funding was received from the Helsinki University Hospital Plastic Surgery Unit. The authors thank Timo Pessi for assistance with statistics; Samu Takala for assistance with video editing; Jukka Alstela and Samu Takala for assistance with photography; and Sirpa Sormunen, the coordinating nurse.

## References

[1] Tuominen R Jahkola T Saisto T Arokoski J Vironen J. The prevalence and consequences of abdominal rectus muscle diastasis among Finnish women: an epidemiological cohort study. Hernia 2022;26:599–608. 34432175 10.1007/s10029-021-02484-8 PMC9012726

[2] El Hawary H Abdelhamid K Meng F Janis JE. A comprehensive, evidence-based literature review of the surgical treatment of rectus diastasis. Plast Reconstr Surg. 2020;146:1151–1164. 33136963 10.1097/PRS.0000000000007252

[3] Emanuelsson P Gunnarsson U Dahlstrand U Strigard K Stark B. Operative correction of abdominal rectus diastasis (ARD) reduces pain and improves abdominal wall muscle strength: a randomized, prospective trial comparing retromuscular mesh repair to double-row, self-retaining sutures. Surgery 2016;160:1367–1375. 27475817 10.1016/j.surg.2016.05.035

[4] Nahas FX Ferreira LM Augusto SM Ghelfond C. Long-term follow-up of correction of rectus diastasis. Plast Reconstr Surg. 2005;115:1736–1741. 15861083 10.1097/01.prs.0000161675.55337.f1

[5] Hickey F Finch JG Khanna A. A systematic review on the outcomes of correction of diastasis of the recti. Hernia 2011;15:607–614. 21688021 10.1007/s10029-011-0839-4

[6] Nahas FX. An aesthetic classification of the abdomen based on the myoaponeurotic layer. Plast Reconstr Surg. 2001;108:1787–1795; discussion 1796. 11711966 10.1097/00006534-200111000-00057

[7] Brauman D. Diastasis recti: clinical anatomy. Plast Reconstr Surg. 2008;122:1564–1569. 18971741 10.1097/PRS.0b013e3181882493

[8] Toranto IR. The relief of low back pain with the WARP abdominoplasty: a preliminary report. Plast Reconstr Surg. 1990;85:545–555. 2138335 10.1097/00006534-199004000-00009

[9] Kohler G Luketina RR Emmanuel K. Sutured repair of primary small umbilical and epigastric hernias: concomitant rectus diastasis is a significant risk factor for recurrence. World J Surg. 2015;39:121–126; discussion 127. 25217109 10.1007/s00268-014-2765-y

[10] Hernández-Granados P Henriksen NA Berrevoet F. European Hernia Society guidelines on management of rectus diastasis. Br J Surg. 2021;108:1189–1191. 34595502 10.1093/bjs/znab128 PMC10364860

[11] Henriksen NA Montgomery A Kaufmann R. Guidelines for treatment of umbilical and epigastric hernias from the European Hernia Society and Americas Hernia Society. Br J Surg. 2020;107:171–190. 31916607 10.1002/bjs.11489

[12] Winocour J Gupta V Ramirez JR Shack RB Grotting JC Higdon KK. Abdominoplasty. Plast Reconstr Surg. 2015;136:597e–606e. 10.1097/PRS.0000000000001700 26505716

[13] Staalesen T Elander A Strandell A Bergh C. A systematic review of outcomes of abdominoplasty. J Plast Surg Hand Surg. 2012;46:139–144. 22747350 10.3109/2000656X.2012.683794

[14] Horlocker TT Vandermeuelen E Kopp SL Gogarten W Leffert LR Benzon HT. Regional anesthesia in the patient receiving antithrombotic or thrombolytic therapy. Reg Anesth Pain Med. 2018;43:263–309. 29561531 10.1097/AAP.0000000000000763

[15] Pollock H Pollock T. Progressive tension sutures: a technique to reduce local complications in abdominoplasty. Plast Reconstr Surg. 2000;105:2583–2586; discussion 2587. 10845315 10.1097/00006534-200006000-00047

